# Distribution of Toxinogenic Methicillin-Resistant and Methicillin-Susceptible *Staphylococcus aureus* from Different Ecological Niches in Algeria

**DOI:** 10.3390/toxins11090500

**Published:** 2019-08-28

**Authors:** Assia Mairi, Abdelaziz Touati, Alix Pantel, Karima Zenati, Alex Yahiaoui Martinez, Catherine Dunyach-Remy, Albert Sotto, Jean-Philippe Lavigne

**Affiliations:** 1 Laboratoire d’Ecologie Microbienne, FSNV, Université de Bejaia, Bejaia 06000, Algeria; 2 National Institute of Health and Medical Research INSERM U1047, University of Montpellier, 30900 Montpellier, France; 3 Department of Microbiology, CHU Nîmes, University of Montpellier, 30900 Montpellier, France; 4 Department of Infectious Diseases, CHU Nîmes, University of Montpellier, 30900 Montpellier, France

**Keywords:** MRSA-ST80, PVL, TSST-1, ecological niches, Algeria, one health, *Staphylococcus aureus*, epidemiology

## Abstract

The diffusion of Panton–Valentine leukocidin (PVL)–positive methicillin-resistant *S. aureus* (MRSA) is a health problem in Algeria. The objectives of the study were to investigate the global distribution of methicillin-susceptible *S. aureus* (MSSA) and MRSA isolates in different ecological niches in this country. In total, 2246 samples were collected from humans, livestock, wild animals, pets, food products and the aquatic environment, from 12 Algerian provinces. A total of 312 *S. aureus* were detected from 2446 samples (12.7%) in the screened niches. We observed the emergence of toxinogenic *S. aureus* representing 41% of the isolates. Among them, we noted the diffusion of ST80-IV CA-MRSA PVL + strains isolated in human, animals, and food and genetic diversity of MSSA PVL + isolates. This study suggests an alarming dissemination of MRSA-ST80 PVL + in both human and extra-human sources in Algeria. Moreover, MSSA may become a permanent reservoir of the PVL genes necessary for human infections.

## 1. Introduction

*Staphylococcus aureus* constitutes a serious public health concern due to its ability to colonize and/or infect humans and animals [1,2,3]. *S. aureus* is the most frequently occurring pathogen in hospitals and the second most common pathogen in outpatient settings [4]. Following the introduction of anti-staphylococcal penicillins, the emergence and diffusion of methicillin-resistant *S. aureus* (MRSA) rapidly appeared in hospitals [1,2]. Successively, a series of predominant clonal strains was characterized. The most important recent clones found in the world are the health-care associated MRSA (HA-MRSA) ST5 and ST239, the community-associated MRSA (CA-MRSA) USA300 and the livestock-associated MRSA (LA-MRSA) ST398. In parts of western Europe, northern Africa and the Middle East, the European ST80 CA-MRSA represents one of the main diffusing clones [1,2].

The pathogenicity of *S. aureus* arises from a large arsenal of virulence factors such as *staphylococcal* enterotoxins (SEs), leukotoxins, hemolysins, exfoliative toxin (ET), toxic shock syndrome toxin-1 (TSST-1) and Panton–Valentine leucocidin (PVL) [5]. The success of some dominant clonal MRSA strains is due to their chromosomal integration of different virulence traits acquired through mobile genetic elements (MGEs) that change the overall fitness of specific clones [6]. Thus, the acquisition of arginine-catabolic mobile element (ACME) has been associated with the spread of USA300 [7]. Moreover, the PVL, a pore-forming cytotoxic secreted toxin encoded in prophage Sa2int, has been associated with severe *S. aureus* pneumonia and prototypical skin lesions [8]. This toxin has been linked to CA-MRSA disease worldwide, even though some of these strains do not carry the PVL genes [9].

In Algeria, the ‘European’ ST80-IV CA-MRSA PVL + clone is widely disseminated in both community and healthcare settings and has given rise to a pandemic clone [2]. Prevalence of ST80-IV CA-MRSA among CA-MRSA isolates varied from 20.7% in Eastern Algeria [10] to 96% in Algiers [11]. However, Algeria and, more widely, Africa has the largest gaps in data on the prevalence of antimicrobial resistance in large reservoirs such as livestock, wild animals or food. While different reports have demonstrated the presence of MRSA in pets, livestock or wild terrestrial or aquatic species [2], no data are available on the prevalence and dissemination of methicillin-sensitive *S. aureus* (MSSA)-PVL + and MRSA-PVL + in extra-human niches, such as farm animals, pets, wild animals, environmental sources, and food products in the same geographical zone [12,13]. The aims of this study were to: (i) estimate the prevalence of *S. aureus* strains recovered in a large collection of samples obtained from different ecological niches distributed among 12 Algerian provinces, (ii) describe the different clones diffusing in these niches and (iii) characterize the genetic contexts of MRSA-PVL + and MSSA-PVL + strains.

## 2. Results

### 2.1. Population of the Isolates

**Figure 1:**
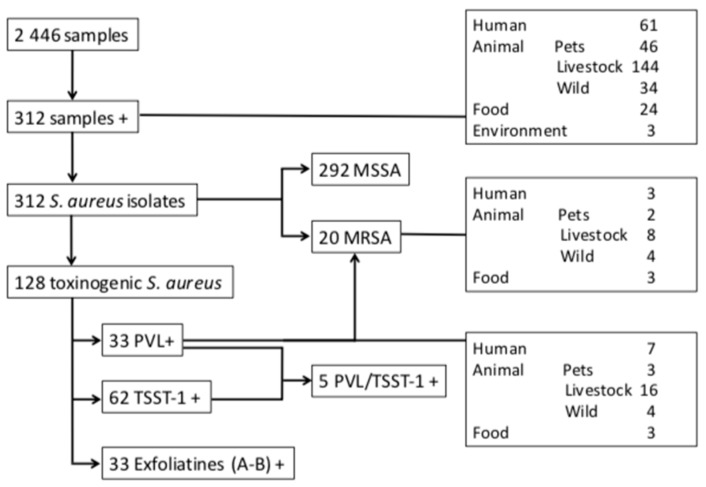
Distribution of the *Staphylococcus aureus* strains isolated from different ecological niches in Algeria.

A total of 312 *S. aureus* isolates obtained from 312 samples were identified giving an overall prevalence of 12.7% (312/2 446) (Figure 1). The isolates were recovered from all the screened niches in the 12 provinces: humans (n = 61; 19.3%), farm animals (n = 144; 18%), pets (n = 46; 14%), wild animal (n = 34; 5.2%), food products (n = 24; 8.6%), and the aquatic environment (n = 3; 4%) (Tables S1 and S2).

### 2.2. Antimicrobial Susceptibility Profiles

The results of the antimicrobial susceptibility of the *S. aureus* isolates are listed in Table 1. Of the 312 *S. aureus* isolates tested, 20 (6.4%) were MRSA harboring *mecA* gene. In contrast, no *mecC* gene was detected. The prevalence of MRSA in the different niches was as follows (Figure 1): farm animals (n = 8/147, 5.4%), wild animals (n = 4/29, 13.7%), food of animal origin (n = 3/22, 13.6%), human (n = 3/61, 4.9%) and pets (n = 2/46, 4.3%). The strains recovered from humans and animals were isolated from nasal swabs (n = 11), feces (n = 3), oral cavity (n = 2) and rectal swab (n = 1). No MRSA strains were detected in environmental samples.

Concerning the co-resistances, most *S. aureus* showed a high prevalence of resistance to penicillin (n = 209, 67%), followed by variable resistance levels to kanamycin (n = 30, 9.6%), erythromycin (n = 24, 7.7%), rifampicin (n = 22, 7%), ofloxacin (n = 11, 3.5%), fusidic acid (n = 8, 2.5%), tobramycin (n = 2, 0.6%), minocycline (n = 1, 0.3%), and clindamycin (n = 1, 0.3%). All isolates were susceptible to ceftobiprole, quinupristin/dalfopristin, gentamicin, fosfomycin and cotrimoxazole. In comparison to MSSA isolates, the 20 MRSA isolates were statistically more frequently resistant to erythromycin (30% vs. 5.4%, *p* = 0.001), ofloxacin (45% vs. 0.6%, *p* < 0.001), fusidic acid (15% vs. 0%, *p* < 0.001), kanamycin (100% vs. 3.7%, *p* < 0.001), and rifampicin (100% vs. 0.6%, *p* < 0.001) and harbored a MLSb phenotype (35% vs. 4.7%, *p* < 0.001). No statistical difference was observed between the different niches.

### 2.3. Importance of Toxinogenic S. Aureus

Of the 312 *S. aureus* isolates recovered from different sources, 128 (41.0%) were toxinogenic with 33 producing PVL (10.6%), 62 TSST-1 (19.8%), and 33 exfoliatins A and B (10.6%) (Figure 1). Among these strains, four co-harbored PVL and TSST-1, one PVL/TSST-1/EdinA, six TSST-1 and an exfoliatin, and 25 PVL + were also EtD + /EdinB + (Table S3).

**Figure 2:**
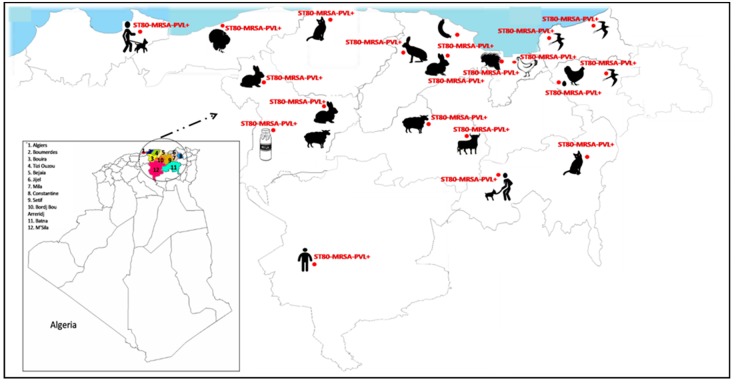
Distribution of the ST80-IV community-associated methicillin-resistant *S. aureus* (CA-MRSA) and the methicillin-susceptible *S. aureus* (MSSA) Panton–Valentine leukocidin (PVL) positive clones circulating in different ecological niches in Algeria.

The PVL + strains were isolated from different sources including animal farms (n = 16, 48.5%), human (n = 7, 21.2%), wild animals (n = 4, 12.1%), cats (n = 3, 9.1%), and food of animal origin (n = 3, 9.1%) (Figure 1 and Figure 2).

The TSST-1 + strains were isolated from humans (n = 18), farm animals (n = 29), pets (n = 13), wild animals (n = 1) and environment (n = 1). Interestingly, the majority of ETs + strains (24/33, 72.7%) were detected from laying hens (5 *etA* + and 19 *etB* +) isolated in different provinces (Table S3). The others were isolated from humans (3 *etA* + and 1 *etB* +), rabbits (2 *etB* +), broilers (1 *etB* +), eggs (1 *etB* +) and dog (1 *etB* +). Finally, the 25 EtD + isolates were PVL +.

**Figure 3:**
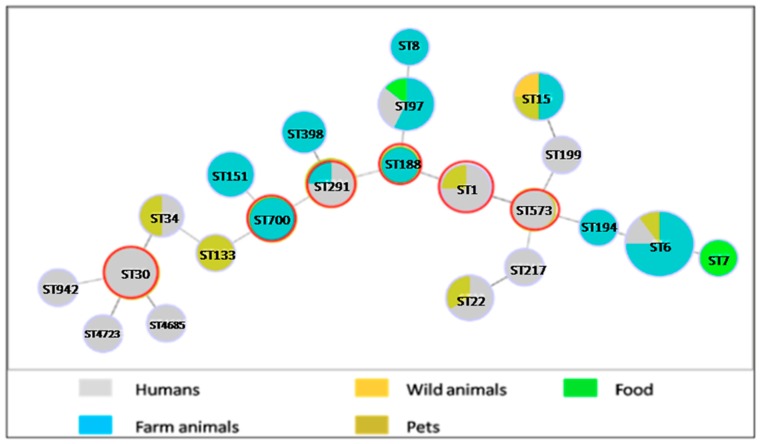
Population snapshot of the methicillin-sensitive *Staphylococcus aureus* MultiLocus Sequence Typing (MLST) dataset generated using the goeBURST algorithm, indicating the sequence types (STs) and their distribution in different sources. The dominant STs are represented by the circles with larger diameters. The founder STs are represented by a red circle.

The Figure 3 shows the goeBURST analysis of the different toxinogenic MSSA-STs identified according to their origin.

Twenty-two STs of this study formed within six groups with ST1, ST30, ST188, ST291, ST573 and ST700 as predicted founders. The formation of four double locus variants (DLV) and seven triple locus variants (TLV) among STs was observed, while no singleton was found. ST6 was the dominant clone (28.5%) recovered among 22 STs identified in this study, distributed in farm animals, humans and pets. The majority of the human isolates were concentrated in ST30 (8.5%), and ST291 (4.2%). Most farm animal isolates were recovered in ST6 followed by ST97, ST151 and ST700. ST133 and ST7 were found in wild animal and food, respectively. Pet isolates were identified in ST15, ST1, ST22 and ST34.

### 2.4. Characteristics of Panton–Valentine Leukocidin (PVL) + S. aureus

Of the 33 PVL + *S. aureus* isolates recovered from different sources, 20 (60.6%) isolates were MRSA and 13 (39.4%) were MSSA. All the MRSA-PVL + isolates belonged to the European ST80-IV CA-MRSA clone and were isolated from humans, animals and food (Figure 1 and Figure 2). The MSSA-PVL + isolates had a more diverse origin: ST6 (n = 7), ST1 (n = 2), ST15 (n = 1), ST398 (n = 1), ST942 (n = 1), and ST97 (n = 1) (Figure 3) and were detected from pets, livestock and humans (Table 2).

The virulence profiles of MRSA and MSSA-PVL + isolates are presented in Table 2. The MRSA-PVL + isolates had some virulence genes significantly associated with their profile compared to MSSA-PVL +. All these isolates harbored the *etD* gene compared to 5 MSSA-PVL + isolates (*p* = 0.005). As the *edinB* gene is on the same operon as *etD*, the same profile was seen for both (*p* = 0.005). Moreover, *agr3* was significantly predominant in MRSA-PVL + isolates (*p* < 0.001). Conversely, the MSSA-PVL + isolates harbored significantly more enterotoxins- (*sea, seh and sei*), leukocidin- (*lukX*), hemolysin- (*hla*), MSCRAMM- (*cna*), capsule- (*cap5*), and an immune evasion cluster- (*chp*) encoding genes. While most of the hemolysins-, leukocidins- and MSCRAMMs-encoding genes were present in both MSSA- and MRSA-PVL + isolates, enterotoxins and *cap5* genes were absent in MRSA-PVL + isolates. No strain harbored the ACME cluster. Finally, the regulator *agr1* gene was significantly predominant in these isolates (*p* < 0.001).

### 2.5. Characteristics of the Other Toxinogenic S. aureus

The main characteristics of virulence profile of the other toxinogenic *S. aureus* isolates are presented in Table 3. We noted that six TSST-1 + isolates harbored *etA* (n = 2) and *etB* (n = 4) genes. The TSST-1 + isolates presented significantly more enterotoxins (seg, seh) compared to non-toxinogenic isolates (*p* < 0.001). These isolates were also significantly associated with the edinB gene (*p* < 0.001) but no etB gene was detected. The seg gene was significantly associated with the ET + isolates (*p* < 0.001).

## 3. Discussion

MRSA isolates remain a worldwide public health problem and have been reported from both hospital and non-hospital reservoirs. In Algeria, the main studies on MRSA have been published from hospital settings. They showed that this resistance mechanism was endemic, involved in a majority of infection and was mainly due to the diffusion of the ST80-IV CA-MRSA clone carrying the PVL genes among the CA-MRSA isolated: 20.7% in Eastern Algeria [10], 73.9% in Western Algeria [14], and 35.7 to 96% in different hospitals of Algiers [11,15,16]. Nasal carriage of this clone has been also described in hospital but with a low prevalence (4/159 enrolled patients) [17]. Finally, the presence of this clone was also found in animals (healthy sheep and camels) [18]. Recently, the CDC proposed the “One Health” concept to organize a worldwide strategy for expanding interdisciplinary collaborations and communications in all aspects of health care for humans, animals and the environment [19]. The authors encouraged prospective prevalence studies to better define the dissemination of the multidrug resistant bacteria, resulting in studies showing the CA-MRSA transmission between livestock and humans especially concerning the ST398 clone [1,2]. Due to the importance of this problem and the unclear and neglected situation of the epidemiology in Algeria, we sought to describe the prevalence, geographic distribution and clonal expansion of MRSA (particularly the ST80-IV CA-MRSA clone) in different ecological niches (human, food, animals, and water) in 12 Algerian provinces. The knowledge of the host specificity and geographic distribution of these bacteria are important parameters for understanding ecology, epidemiology, and diffusion of this resistance gene. Therefore, our study has established, for the first time to our knowledge, a valuable identification of the ST80-IV CA-MRSA isolates recovered from different sources and their virulence profiles.

Across a wide range of different reservoirs screened, it is interesting to note that ST80-IV CA-MRSA PVL + was the single methicillin-resistant clone found in our study, representing an overall prevalence of 6.4% of all the environments screened (except aquatic environment). This wide diffusion is of importance notably due to the presence of this clone in niches other than humans. This highlights the worrisome situation of multidrug resistance in this country. Our results also show the dynamic capacity of adaptation of the ST80-IV CA-MRSA isolates, and highlight that farm animals, companion animals, wildlife, food, etc. may become a permanent reservoir for human infections. The hospital environment is a visible threat for this country, according to the main studies, however, the community is not spared. Several practices allow the spread of hospital isolates to the community, and vice versa [20]. Therefore, even though control measures can be taken at the hospital, the eradication of this clone will be nearly impossible due to its presence in the community. Surveillance programs outside the hospital will need to be implemented to control its spread.

Although *pvl* genes were associated with ST80-IV CA-MRSA, our results also showed that some MSSA PVL + isolates emerged from different sources. While the distribution of MSSA PVL + clones is scarcely described in Algeria, it is important to quantify their prevalence because these strains are frequently associated with skin and soft-tissue infections in Algerian hospitals. Their potential to cause infections in humans through the food chain similarly needs attention. Six STs of MSSA-PVL + were found (ST1, ST6, ST15, ST97, ST398, and ST942), mainly from farm animals. All of these STs have been previously described by Agabou et al. in Algeria [18]. If ST15-PVL + MSSA is a very common worldwide strain reported in many countries [21,22,23], the ST6-PVL + MSSA was more unusual and has previously been identified in reports from Africa, as well as from China [22,24,25].The ST97-PVL + MSSA has been reported only in one human case from Bali [26]. The ST398 is frequently isolated from pigs [1,2,27] and the ST97 is globally linked with bovine mastitis [2,28,29]. In addition, all these types of MSSA have been reported from a variety of clinical infections [30,31,32,33,34].

One of the other main findings in our study is the high prevalence of toxinogenic MSSA strains isolated from different niches screened, in particular the TSST-1 + MSSA isolates (19.8%). This virulence factor is rare in human in Algeria [15,35] but was previously described in farm animals and food product [18,36]. Twenty-one MSSA TSST-1 + clones were distributed across different sources comprising a wide range of clones. These findings demonstrated a large diversity among strains obtained from different sources. The *S. aureus* pathogenicity islands containing the *tst* gene are classically mobilized in high frequency following infection by some bacteriophages [37]. Using the PHYLOVIZ algorithm, which provides a hypothetical pattern of descent for the analyzed isolates, we concluded that most of the PVL + and TSST-1 + MSSA isolates of this study formed different clonal complexes, suggesting a high genotypic diversity. ST6 was found to be the predominant ST representing about 23.5% of the total STs, which was recovered in farm animals, humans and pets suggesting that the strain may be transmissible across different host species. This clone had previously been reported from humans (MSSA and MRSA), feral cat (MRSA) and camel (MSSA) [18,38,39]. ST30 was only recovered from human isolates [40]. The clustered MSSA isolates included in this study demonstrated different clone variations circulating from humans, farm animal and pets.

Interestingly, *S. aureus* can generate genetic variations in its genome mainly by horizontal gene transfer enabling it to adapt rapidly to new ecological niches [6,7]. The introduction of new gene combinations may play an important role in the increase of the pathogenicity or the diffusion of the bacterium and in the better adaptation to new hosts [2]. Here, the presence of some genes in strains isolated from animals suggests a human origin. For example, *chp*, *ebh*, *fnbA*, *hlb*, *sak*, *scn*, *sea* genes have been associated with a human specificity and their absence may be a valuable indicator of *S. aureus* animal adaptation [41]. We observed this trend with a low prevalence of these markers in the animal isolates (16/224 for *fnbA*, 91/224 for *sea* or 25/224 for *hlb*). Moreover, *fnbA*, *ebh*, *sak*, *hlb* (15/20 isolates) and *scn* (17/20 isolates) genes were detected in ST80-IV CA-MRSA isolates, which confirms their human origin [42]. Finally, the detection of the *edinB* and *etD* genes in these MRSA isolates illustrates the dissemination of toxinogenic strains with multiple profiles (PVL +, TSST-1 +, EdinB + and EtD +) and a high pathogenicity potential relevant for human and animal health. This also demonstrates the high potential of MGEs such as phages (for *lukS-PV* and *lukF-PV*) or pathogenicity islands (for *tst*) to appear in a single isolate, to reach different STs (3 for 5 isolates) and to diffuse in different environments (ovine, cat, horse owner, farmer) suggesting that bacteriophages could have originally allowed the spread of these genes among lineages, as previously hypothesized [43]. The combination of major virulence factors in the same strain must be further studied. Their persistence and expansion will depend on the fitness cost for the bacteria [44]. Thus, the presence of these multiple toxinogenic genes in *S. aureus* isolated in different ecosystems may pose a public health risk by convergences between habitats leading to frequent contact between animals, environment and humans.

## 4. Conclusions

This study suggests a high prevalence and a wild diffusion of toxinogenic *S. aureus* isolates and especially the ST80-IV CA-MRSA clones among different niches in 12 Algerian provinces. This wide dissemination in different niches other than the clinical setting may constitute a reservoir of resistant strains, which could be responsible for transmission from humans to their cohabitants and within veterinary hospitals. This dissemination is certainly due to the excessive use of antimicrobial agents and the presence of antibiotics in different environments (animals, food, soil, waste water) as previously suggested [45], along with insufficient infection control measures, and further studies are required to elucidate the cycle of transmission of these bacteria between these different habitats and to explore the origin of MRSA isolates circulating outside hospitals. This is of importance due to the high potential of virulence of these isolates.

## 5. Materials and Methods

### 5.1. Sampling and Microbiological Procedures

The study was approved by the local Ethical Committee (Béjaia, Algeria). The approval date is 17 October 2017. The humans included in this study gave their consent. From January to July 2018, different randomly and prospectively selected niches were chosen, localized in 12 provinces in Algeria (Bejaia, Tizi Ouzou, Bouira, Jijel, Mila, Setif, Algiers, Boumerdes, Constantine, Batna, M’Sila and Bordj Bou Arreridj). This large group of samples was then randomized for testing. A total of 2446 samples were randomly collected, including samples from humans (community, n = 100; pet’ owners, n = 181 and farmers, n = 35), farm animals (n = 800), pets (n = 328), wild animals (n = 652), food products (n = 276) and water environment (n = 74) (Tables S1 and S2).

Humans and domestic animals screening was by nasal swabs, whilst wild animals and fish were screened via fresh fecal droppings and intestinal content samples, respectively. Poultry were screened for *S. aureus* buccal and rectal carriage. Local food products (chicken, meat, sausage, milk and eggs) were randomly obtained from different markets, stores and farms of the 12 studied provinces. Samples of aquatic environment were collected from seawater (Mediterranean Sea, n = 22), spring water (n = 16), rivers (n = 5), lakes (n = 7), dam water (n = 12) and fountains (n = 12). One hundred milliliters of milk and water samples were taken in sterile flasks.

All samples were immediately transported at + 4 °C to the Microbiological Ecology Laboratory at the University of Bejaia (Algeria) for analysis. They were processed within the day after sampling. The samples were cultured in trypticase soy broth (TSB) (Fluka, St Louis, MO, USA) supplemented with colistin (10 mg/L), aztreonam (10 mg/L) and a mphotericin B (2 mg/L) and incubated for 18 h at 37 °C. For food products, 25 g of each food item or 25 mL of milk were cultured in 225 mL of TSB supplemented with the same antibiotics as described above and incubated for 18 h at 37 °C. Following incubation, a 200 μL aliquot was plated onto mannitol salt agar (Fluka, St Louis, MO, USA) incubated for 24 and 48 h at 37 °C. For water, the procedure of isolation consisted on inoculation of 200 μL aliquot of 1:10 diluted samples on mannitol salt agar.

Presumptive isolates were sub-cultured on TSA and identified as *S. aureus* by the Vitek MS® system (bioMérieux, Marcy l’Etoile, France).

### 5.2. Antibiotic Susceptibility Testing

Susceptibility to 14 antimicrobial agents (penicillin G, cefoxitin, erythromycin, clindamycin, quinupristin/dalfopristin, kanamycin, tobramycin, gentamicin, minocycline, ofloxacin, fusidic acid, fosfomycin, rifampicin and cotrimoxazole) was tested by the disk diffusion method on Mueller Hinton agar (BioRad, Marnes La Coquette, France) according to recommendations of EUCAST 2018 [46]. A cefoxitin disk was used to screen the MRSA isolates. To confirm the presence/absence of *mecA* and *mecC* genes, we performed PCR as previously described [47].

### 5.3. DNA Arrays Procedures

All the *S. aureus* strains isolated during the study were then analyzed by Alere Staphy Type DNA microarray according to protocols and procedures previously detailed [38,48]. This array simultaneously detects 333 *S. aureus* target sequences, including species markers, antimicrobial resistance and virulence-associated genes, and SCC*mec*-associated genes and typing markers, allowing isolates to be assigned to MultiLocus Sequence Typing (MLST) sequence types (STs), clonal complexes (CCs) and SCC*mec* types. Primer and probe sequences have been previously published [48]. Raw data were interpreted as “positive”, “negative” or “ambiguous” using a previously described algorithm [48]. The affiliation of isolates to CCs or STs as defined by MLST was determined by an automated comparison of hybridization profiles with a collection of reference strains previously characterized [49].

### 5.4. MultiLocus Sequence Typing (MLST)

To confirm array results, MLST analysis was performed on the toxinogenic MSSA isolates as previously described [50]. Seven housekeeping genes (*arc*, *aroE*, *glpF*, *gmk*, *pta*, *tpi* and *yqi*) were sequenced to determine allelic profile. Strains were assigned to a ST using the MLST database [51].

### 5.5. goeBURST Analysis of Methicillin-Susceptible S. Aureus (MSSA) Distribution

In order to determine the distribution of STs of toxinogenic MSSA strains isolated from different sources, MLST dataset was performed using goeBURST software [52] implemented by the PHYLOVIZ program [53] available at http://phyloviz.net. Related ST is inserted in the software to provide an unrooted tree-based representation of the relationship of the founding genotype, reflected in the appearance of STs differing only in one housekeeping gene sequence from the founder genotype-single locus variants (SLVs). Further diversification of those SLVs results in the appearance of variations of the original genotype with more than one difference in the allelic profile: double locus variants (DLVs), triple locus variants (TLVs) and so on.

### 5.6. Statistical Analysis

Data are displayed as frequencies (effective, %) and pairwise comparisons between the resistance profile of MSSA and MRSA isolates, MRSA-PVL + and MSSA-PVL + isolates and toxinogenic and non-toxinogenic isolates were assessed by a chi-square test or Fisher’s exact two-tailed test (when n < 5) using the statistical software GraphPad Instat Prism vers.6.04 (GraphPad Software Inc., San Diego, CA, USA. 2014). Statistical significance was set at a *p* ≤ 0.05.

## Figures and Tables

**Table 1:** Resistance profiles of *S. aureus* strains isolated from different niches in 12 Algerian provinces*.

Antibiotics	Origin of Isolates										
	Human	Bovine	Ovine	Goats	Rabbits	Poultry	Pets	Wild Animals	Food Products	Aquatic Environment	Total Samples
Total	61	18	34	8	15	69	46	34	24	3	312
Penicillin G	51 (83.6)	11 (61.1)	19 (55.8)	4 (50)	8 (53.3)	51 (73.9)	28 (60.8)	25 (73.5)	11 (45.8)	1 (33.3)	209 (66.9)
Cefoxitin	3 (4.9)	1 (5.5)	1 (2.9)	0 (0)	3 (20)	3 (4.3)	2 (4.3)	5 (14.7)	2 (8.3)	0 (0)	20 (6.4)
Erythromycin	5 (8.1)	0 (0)	1 (2.9)	0 (0)	0 (0)	6 (8.6)	4 (8.6)	2 (5.8)	6 (25)	0 (0)	24 (7.6)
Ceftobiprole	0 (0)	0 (0)	0 (0)	0 (0)	0 (0)	0 (0)	0 (0)	0 (0)	0 (0)	0 (0)	0 (0)
Clindamycin	1 (1.6)	0 (0)	0 (0)	0 (0)	0 (0)	0 (0)	0 (0)	0 (0)	0 (0)	0 (0)	1 (0.3)
Quinupristin/Dalfopristin	0 (0)	0 (0)	0 (0)	0 (0)	0 (0)	0 (0)	0 (0)	0 (0)	0 (0)	0 (0)	0 (0)
Kanamycin	4 (6.5)	3 (16.6)	4 (11.7)	0 (0)	3 (20)	4 (5.7)	2 (4.3)	5 (14.7)	4 (16.6)	1 (33.3)	30 (9.6)
Tobramycin	0 (0)	0 (0)	0 (0)	0 (0)	0 (0)	0 (0)	0 (0)	0 (0)	2 (8.3)	0 (0)	2 (0.6)
Gentamicin	0 (0)	0 (0)	0 (0)	0 (0)	0 (0)	0 (0)	0 (0)	0 (0)	0 (0)	0 (0)	0 (0)
Minocycline	0 (0)	0 (0)	0 (0)	0 (0)	0 (0)	1 (1.4)	0 (0)	0 (0)	0 (0)	0 (0)	1 (0.3)
Ofloxacin	2 (3.2)	0 (0)	0 (0)	0 (0)	2 (13.3)	1 (1.4)	1 (2.1)	3 (8.8)	2 (8.3)	0 (0)	11 (3.5)
Fusidic acid	1 (1.6)	0 (0)	0 (0)	0 (0)	0 (0)	1 (1.4)	0 (0)	5 (14.7)	1 (4.1)	0 (0)	8 (2.5)
Fosfomycin	0 (0)	0 (0)	0 (0)	0 (0)	0 (0)	0 (0)	0 (0)	0 (0)	0 (0)	0 (0)	0 (0)
Rifampicin	3 (4.9)	1 (5.5)	1 (2.9)	0 (0)	3 (20)	3 (4.3)	2 (4.3)	7 (20.5)	2 (8.3)	0 (0)	22 (7.0)
Cotrimoxazole	0 (0)	0 (0)	0 (0)	0 (0)	0 (0)	0 (0)	0 (0)	0 (0)	0 (0)	0 (0)	0 (0)

*Values indicate the number of isolates tested with the percentage of resistance in parenthesis (%).

**Table 2:** Distribution of the main virulence and resistance genes among the *S. aureus* PVL + isolated from different ecological niches in Algeria.

Virulence and Resistance Genes	MRSA-PVL + no. (%)	MSSA-PVL + no. (%)	*p* Value
Virulence Genotyping			
Enterotoxins			
*sea*	0 (0)	8 (61.5)	< 0.001
*seb*	0 (0)	2 (15.3)	NS
*seg*	0 (0)	2 (15.3)	NS
*seh*	0 (0)	3 (23)	0.05
*sei*	0 (0)	5 (38.4)	0.005
*sek*	0 (0)	1 (7.6)	NS
*seq*	0 (0)	1 (7.6)	NS
*egc cluster^a^*	0 (0)	0 (0)	NS
Other Toxins			
*tst*	0 (0)	5 (38.4)	0.005
*etA*	0 (0)	0 (0)	NS
*etB*	0 (0)	0 (0)	NS
*etD*	20 (100)	5 (38.4)	0.005
*edinA*	0 (0)	1 (7.6)	NS
*edinB*	20 (100)	5 (38.4)	0.005
Leukocidins			
*lukD*	20 (100)	12 (92.3)	NS
*lukE*	19 (95)	12 (92.3)	NS
*lukX*	7 (35)	13 (100)	< 0.001
*lukY*	15 (75)	13 (100)	NS
Hemolysins			
*hla*	10 (50)	12 (92.3)	0.02
*hlb*	15 (75)	6 (46.1)	NS
*hld*	20 (100)	13 (100)	NS
*hlgA*	20 (100)	13 (100)	NS
*hlgv*	20 (100)	13 (100)	NS
Microbial Surface Components Recognizing Adhesive Matrix Molecules (MSRAMMs)			
*bbp*	20 (100)	11 (84.6)	NS
*clfA*	20 (100)	13 (100)	NS
*clfB*	20 (100)	13 (100)	NS
*cna*	0 (0)	5 (38.4)	0.005
*ebpS*	20 (100)	13 (100)	NS
*fib*	20 (100)	12 (92.3)	NS
*fnbA*	20 (100)	12 (92.3)	NS
*fnbB*	20 (100)	13 (100)	NS
Capsule Components			
*cap5*	0 (0)	3 (23)	0.05
*cap8*	20 (100)	10 (76.9)	NS
Intracellular Adhesion Polysaccharide			
*icaA*	20 (100)	13 (100)	NS
*icaC*	20 (100)	13 (100)	NS
*icaD*	20 (100)	13 (100)	NS
Immune Evasion Cluster and Other			
*sak*	20 (100)	10 (76.9)	NS
*chp*	0 (0)	3 (23)	0.05
*scn*	17 (85)	11 (84.6)	NS
ACME cluster^b^	0 (0)	0 (0)	NS
Accessory Gene Regulators			
*agr1*	0 (0)	9 (69.2)	< 0.001
*agr2*	0 (0)	1 (7.6)	NS
*agr3*	20 (100)	3 (23)	< 0.001
*agr4*	0 (0)	0 (0)	NS
Resistance Genotyping			
*mecA*	20 (100)	0 (0)	< 0.001
*mecC*	0 (0)	0 (0)	NS
*blaZ*	2 (10)	10 (76.9)	< 0.001
*ermA*	0 (0)	0 (0)	NS
*ermC*	3 (15)	0 (0)	NS
*aphA3*	20 (100)	1 (7.6)	< 0.001
*sat*	20 (100)	1 (7.6)	< 0.001
*fosB*	1 (5)	3 (23)	NS

^a^*egc* cluster corresponds to *seg, sei, sem, sen* and *seo* genes; ^b^ ACME (arginine catabolic mobile element) includes the arc genes (*arcA* to *arcD*) and the oligopeptide permease operon genes (*opp-3A* to *opp-3E*). NS, not significant (*p* > 0.05).

**Table 3:** Distribution of the main virulence genes among the toxinogenic and non toxinogenic *S. aureus* (excluded PVL +) isolated from different ecological niches in Algeria.

Virulence and Resistance Genes	TSST-1 + no. (%)	Et + no. (%)	Non Toxinogenic MSSA no. (%)	*p* value^a^
Isolates	n = 62	n = 33	n = 203	
Enterotoxins				
*sea*	36 (58.0)	26 (78.7)	115 (56.6)	NS
*seb*	7 (11.2)	2 (6.0)	14 (6.8)	NS
*seg*	43 (69.3)	30 (90.9)	38 (18.7)	< 0.001
*seh*	18 (29.0)	0 (0)	16 (7.8)	0.001
*sei*	62 (100)	32 (96.9)	179 (88.1)	NS
*sek*	13 (20.9)	2 (6.0)	20 (9.8)	NS
*seq*	13 (20.9)	2 (6.0)	16 (7.8)	NS
Other Toxins				
*tst*	62 (100)	6 (18.1)	0 (0)	< 0.001
*etA*	2 (3.2)	8 (24.2)	0 (0)	< 0.001
*etB*	4 (6.4)	25 (75.7)	0 (0)	< 0.001
*etD*	0 (0)	0 (0)	0 (0)	NS
*edinA*	0 (0)	0 (0)	0 (0)	NS
*edinB*	24 (38.7)	1 (3.0)	18 (8.8)	< 0.001
Leukocidins				
*lukD*	62 (100)	33 (100)	203 (100)	NS
*lukE*	62 (100)	33 (100)	203 (100)	NS
Hemolysins				
*hla*	55 (88.7)	33 (100)	191 (94.0)	NS
*hlb*	24 (38.7)	1 (3.0)	43 (21.1)	NS
*hld*	62 (100)	33 (100)	203 (100)	NS
*hlgA*	49 (79.0)	33 (100)	188 (92.6)	NS
*hlgv*	60 (96.7)	32 (96.9)	197 (97.0)	NS
MSCRAMMs				
*bbp*	62 (100)	33 (100)	203 (100)	NS
*clfA*	62 (100)	33 (100)	203 (100)	NS
*clfB*	62 (100)	33 (100)	203 (100)	NS
*cna*	0 (0)	0 (0)	14 (6.8)	0.006
*ebpS*	62 (100)	33 (100)	203 (100)	NS
*fib*	55 (88.7)	32 (96.9)	187 (92.1)	NS
*fnbA*	10 (16.1)	7 (21.2)	26 (12.8)	NS
*fnbB*	28 (45.1)	6 (18.1)	62 (30.5)	NS
Capsule Components				
*cap5*	35 (56.5)	20 (60.6)	138 (68.0)	NS
*cap8*	27 (44.5)	13 (39.4)	65 (32.0)	NS
Intracellular Adhesion Polysaccharide				
*icaA*	62 (100)	33 (100)	203 (100)	NS
*icaC*	62 (100)	33 (100)	203 (100)	NS
*icaD*	62 (100)	33 (100)	203 (100)	NS
Other				
ACME cluster^b^	0 (0)	0 (0)	0 (0)	NS
Accessory Gene Regulators				
*agr1*	42 (67.7)	25 (75.7)	153 (75.3)	NS
*agr2*	6 (9.6)	3 (9.0)	31 (15.2)	NS
*agr3*	14 (22.5)	2 (6.0)	19 (9.3)	NS
*agr4*	0 (0)	3 (9.0)	0 (0)	NS

^a^*p* value corresponds to comparison between toxinogenic and non toxinogenic *S. aureus* isolates using Fisher exact test. ^b^ ACME (arginine catabolic mobile element) includes the arc genes (*arcA* to *arcD*) and the oligopeptide permease operon genes (*opp-3A* to *opp-3E*). NS, not significant (*p* > 0.05).

## Supplementary Materials

The following are available online at https://www.mdpi.com/2072-6651/11/9/500/s1: Table S1: Numbers and distribution of samples collected from different ecological niches in 12 Algerian provinces, Table S2: Numbers and distribution of samples in the different ecological niches, Table S3: Characteristics of MSSA strains isolated from different niches in Algeria.
