# Supplementary material for: Distribution of Toxinogenic Methicillin-Resistant and Methicillin-Susceptible Staphylococcus aureus from Different Ecological Niches in Algeria
Source: Toxins (Basel). 2019 Aug 28;11(9):500. doi: 10.3390/toxins11090500 (PMC6784135; doi:10.3390/toxins11090500)
Supplement: Supplementary file 1 [file toxins-11-00500-s001.pdf]

Supplementary Materials: Distribution of Toxinogenic Methicillin-Resistant and Methicillin-Susceptible Staphylococcus aureus From Different Ecological Niches in Algeria

Assia Mairi, Abdelaziz Touati, Alix Pantel, Karima Zenati, Alex Yahiaoui Martinez, Catherine Dunyach-Remy, Albert Sotto and Jean-Philippe Lavigne

Table S1. Numbers and distribution of samples collected from different ecological niches in 12 Algerian provinces.

Provinces	Samples Collected (n)	*Staphylococcus aureus* (n)
**Bejaia**	770	155
**Bordj Bou Arreridj**	194	21
**Bouira**	169	16
**Jijel**	135	14
**Mila**	145	11
**Tizi Ouzou**	173	18
**Setif**	145	11
**Alger**	200	19
**Boumerdes**	124	10
**M'Sila**	151	13
**Constantine**	143	10
**Batna**	97	14
**Total**	2446	312

Table S2. Numbers and distribution of samples in the different ecological niches in Algeria.

Niches	Samples Collected (n)	*Staphylococcus aureus* (n)
Animals	1780	224
Farm animals (nasal samples)	800	144
Bovine	196	18
Broilers	126	4
Goats	67	8
Laying hens	141	59
Ovine	184	34
Rabbits	41	15
Turkeys	45	6
Pets (nasal samples)	328	46
Cats	90	17
Dogs	149	23
Horses	89	6
Wild animals (fecal samples)	652	34
Wild birds	237	18
Wild fish	182	6
Barbary macaques	65	1
Wild Boars	65	5
Barbarous deer	37	0
Jackal	16	0
Hares	27	2
Wolves	10	0
Porcupine	13	2

Food products (fresh samples)	276	24
Chicken carcasses	20	0
Chicken offal's	45	2
Goat milk	32	2
Cow milk	46	7
Meat	49	4
Sausages	24	2
Table eggs	60	7
Human (nasal samples)	316	61
Pets owners	181	24
Farmers	35	12
Volunteers in community	100	25
Aquatic environment (water)	74	3
Total	2446	312

Table S3. Characteristics of MSSA strains isolated from different niches in Algeria.

Strain	Origin	Date of Sampling	Sampling Type	Localisation	AMR Profiles	PVL Content	TSST Content	Et Content	Other Toxin Genes Detected	*agr type*	MLST
S46	Bovine (*Bos taurus*)	22/04/2018	Nasal	Bourdj Bou Arreridj (Khelil)	-	+	-	-	*sea, seb, sei, hla, hld, hlg, hlg2, clfB, fib*	1	ST6
S47	Bovine (*Bos taurus*)	06/02/2018	Nasal	Bejaia (Boukhelifa)	PNG1-KMN	+	-	-	*seb, hla, hld, hlg, hlg2, clfB, fib*	1	ST97
S77	Ovine (*Ovis aries*)	11/07/2018	Nasal	Bouira	PNG1	+	+	-	*sea, hla, hld, hlg, hlg2, clfB, fib, fnbA, fnbB*	1	ST6
S78	Ovine (*Ovis aries*)	01/02/2018	Nasal	Setif	PNG1	+	-	-	*hla, hld, hlg, hlg2, clfB, fnbA, fnbB*	1	ST398
S155	Laying hens	02/05/2018	Oral	Tizi Ouzou	PNG1	+	-	-	*sea, sei, clfB, fib, fnbA, fnbB, edinB, hla, hld, hlg, hlg2*	1	ST6
S156	Laying hens	22/04/2018	Oral	Bourdj Bou Arreridj (Khelil)	PNG1	+	-	-	*sea, sei, clfB, fib, fnbA, fnbB, edinB, hla, hld, hlg, hlg2*	1	ST6
S157	Laying hens	16/04/2018	Rectal	Bejaia (Timezrit)	PNG1	+	-	-	*sea, sei, clfB, fib, fnbA, fnbB, edinB, hla, hld, hlg, hlg2*	1	ST6
S158	Laying hens	18/04/2018	Rectal	Bejaia (Beni Ksila)	-	+	-	-	*clfB, fib, fnbA, fnbB, hla, hld, hlg, hlg2*	2	ST15
S181	Cat	04/02/2018	Nasal	Bejaia	PNG1	+	+	-	*sea, seg, seh, sei, hla, hld, hlg, hlg2, clfB, fib, fnbA, fnbB, edinB*	1	ST6
S215	Horse owner	18/02/2018	Nasal	Constantine	PNG1-FAD	+	+	-	*sea, seh, sek, seq, hla, hlb, hld, hlg, hlg2, clfB, fib, cna, fnbA, fnbB*	3	ST1
S242	Farmer	26/04/2018	Nasal	Bejaia (Akbou)	PNG1	+	-	-	*sea, hla, hld, hlg, hlg2, clfB, fib, fnbA, fnbB*	1	ST6
S243	Farmer	02/03/2018	Nasal	Algiers (Baraki)	PNG1	+	+	-	*hla, hld, hlg, hlg2, clfB, fib, fnbA, fnbB*	3	ST1
S244	Farmer	11/07/2018	Nasal	Bouira	PNG1	+	+	-	*hld, hlg, hlg2, clfB, fib, fnbB*	3	ST942
S30	Hare (*Lepus capensis*)	20/06/2018	Feces	Bejaia (Akfadou)	PNG1	-	+	-	*fnbB, clfB, fib, sea, sek, sei, seg, seq, seh, hla, hld, hlg, hlg2*	2	ST15
S31	Bovine (*Bos taurus*)	13/02/2018	Nasal	Bejaia (Beni Maouche)	-	-	+	-	*clfB, fib, sec, sei, seg, hla, hlb, hld, hlg, hlg2*	1	ST188
S38	Bovine (*Bos taurus*)	13/02/2018	Nasal	Bejaia (Beni Maouche)	-	-	+	-	*fnbA, clfB, fib, sec, sei, seg, hla, hld, hlg2*	2	ST151
S51	Ovine (*Ovis aries*)	22/04/2018	Nasal	Bourdj Bou Arreridj (Zemoura)	-	-	+	-	*fnbB, clfB, fib, sea, sec, sei, edin, hla, hlb, hld, hlg*	1	ST700
S54	Ovine (*Ovis aries*)	11/07/2018	Nasal	Bouira	PNG1	-	+	-	*fnbB, clfB, fib, sea, sec, sei, edin, hld, hlg, hlg2*	1	ST291
S56	Ovine (*Ovis aries*)	24/04/2018	Nasal	Mila	PNG1	-	+	-	*fnbB, clfB, fib, seb, sec, sei, edinB, hla, hld, hlg, hlg2*	1	ST97

S57	Ovine (*Ovis aries*)	01/02/2018	Nasal	Setif	PNG1	-	+	-	*fnbA, clfB, fib, sec, sei, seg, edinB, hla, hld, hlg, hlg2*	2	ST15
S60	Ovine (*Ovis aries*)	12/02/2018	Nasal	Bejaia (Ighezar Amokrane)	PNG1	-	+	-	*fnbB, clfB, fib, sec, sei, hla, hlb, hld, hlg, hld2*	1	ST8
S62	Ovine (*Ovis aries*)	30/06/2018	Nasal	Mila	PNG1	-	+	-	*fnbB, clfB, fib, sec, sei, seg, edinB, hla, hlb, hld, hlg*	2	ST151
S63	Ovine (*Ovis aries*)	24/04/2018	Nasal	Mila	-	-	+	-	*clfB, fib, sec, sei, seh, edinB, hla, hlb, hld, hlg2*	3	ST700
S65	Ovine (*Ovis aries*)	06/02/2018	Nasal	Bejaia (Boukhelifa)	PNG1	-	+	-	*fnbB, clfB, fib, seb, sei, seg, hla, hlb, hld, hlg, hlg2*	1	ST6
S73	Ovine (*Ovis aries*)	17/02/2018	Nasal	Bousaada	-	-	+	-	*clfB, fib, sec, sei, edinB, hla,hlb, hld, hlg2*	3	ST700
S74	Ovine (*Ovis aries*)	17/02/2018	Nasal	Bousaada	-	-	+	-	*fnbA, clfB, fib, seb, sec, sei, seg, hla, hlb, hld, hlg, hlg2*	1	ST6
S75	Ovine (*Ovis aries*)	06/02/2018	Nasal	Bejaia (Boukhelifa)	-	-	+	-	*clfB, fib, sec, sei, seg, hla, hlb, hld, hlg, hlg2*	1	ST6
S76	Ovine (*Ovis aries*)	12/02/2018	Nasal	Bejaia (Sidi Aich)	-	-	+	-	*fnbA, fnbB, clfB, fib, sec, sei, seg*	3	ST151
S79	Goat (*Capra hircus*)	11/02/2018	Nasal	Bejaia (Sidi Aich)	-	-	+	-	*fnbB, clfB, fib, sea, sei, edinB, hla, hld, hlg, hlg2*	1	ST97
S80	Goat (*Capra hircus*)	24/05/2018	Nasal	Jijel	-	-	+	-	*clfB, fib, sea, sec, sei, edinB, hla, hlb, hld, hlg, hlg2*	1	ST6
S88	Goat (*Capra aegagrus hircus*)	02/05/2018	Nasal	Tizi Ouzou	-	-	+	-	*fnbA, clfB, fib, sei, seg, hla, hlb, hld, hlg, hlg2*	1	ST6
S92	Rabbit	25/04/2018	Nasal	Bejaia (Barbacha)	PNG1	-	+	-	*fnbB, clfB, fib, sea, sec, sei, edinB, hla, hld, hlg, hlg2*	1	ST194
S94	Rabbit	09/02/2018	Nasal	Bejaia (Ouzellaguen)	PNG1	-	+	+	*clfB, fib, etb, sea, sec, sei, edinB, hla, hld, hlg, hlg2*	1	ST6
S117	Laying hens	10/02/2018	Rectal	Bejaia (Tazmalt)	PNG1	-	+	+	*fnbB, clfB, fib, eta, sea, sek, sei, seg, sek, hla, hld, hlg, hlg2*	1	ST398
S135	Laying hens	16/04/2018	Rectal	Bejaia (Timezrit)	PNG1	-	+	+	*clfB, fib, etb, sea, sei, seg, hla, hld, hlg, hlg2*	1	ST97
S138	Laying hens	16/04/2018	Oral	Bejaia (Timezrit)	PNG1	-	+	-	*fnbA, clfB, fib, sea, sei, seg, edinB, hla, hld, hlg, hlg2*	1	ST6
S145	Laying hens	22/04/2018	Oral	Bourdj Bou Arreridj (Khelil)	-	-	+	+	*fnbA, clfB, fib, etb, sea, sei, seg, hla, hld, hlg, hlg2*	1	ST6
S152	Laying hens	19/05/2018	Oral	Batna	-	-	+	-	*fnbB, clfB, fib, sea, sek, sei, seg, seq, seh, hla, hlb, hld, hlg, hlg2*	1	ST6
S173	Cat	10/05/2018	Nasal	Boumerdes	PNG1	-	+	-	*clfB, sei, seg, hla, hld, hlg2*	2	ST22
S187	Dog	21/02/2018	Nasal	Bejaia	PNG1	-	+	-	*fnbB, clfB, fib, sea, sei, seg, sed, edinB, hla, hlb, hld, hlg, hlg2*	1	ST15

S194	Dog	21/04/2018	Nasal	Bejaia (El Kseur)	-	-	+	-	*clfB, fib, sea, sek, sei, seg, seq, seh, hla, hlb, hld, hlg, hlg2*	1	ST6
S200	Dog	01/05/2018	Nasal	Tizi Ouzou	PNG1	-	+	-	*clfB, sea, sei, seg, sed, hld, hlg2*	3	ST34
S206	Horse	18/02/2018	Nasal	Constantine	PNG1	-	+	-	*clfB, fib, sea, sek, sei, seg, seq, seh, hla, hlb, hld, hlg, hlg2*	3	ST1
S209	Horse	07/03/2018	Nasal	Algiers (Bouchaoui)	-	-	+	-	*clfB, fib, sea, sek, sei, seh, hla, hld, hlg, hlg2*	1	ST133
S213	Horse owner	07/05/2018	Nasal	Bejaia (Adekar)	-	-	+	-	*fnbA, clfB, fib, sea, sei, seh, hla, hlb, hld, hlg, hlg2*	1	ST573
S214	Horse owner	01/02/2018	Nasal	Setif	-	-	+	-	*fnbB, clfB, sei, seg, edinB, hld, hlg, hlg2*	1	ST291
S216	Cat owner	04/02/2018	Nasal	Bejaia	PNG1	-	+	-	*fnbB, clfB, fib, sea, sek, sei, seg, seq, seh, edinB, hla, hlb, hld, hlg, hlg2*	3	ST291
S217	Cat owner	21/02/2018	Nasal	Bejaia	PNG1	-	+	-	*fnbB, clfB, fib, sea, sek, sei, seg, seq, seh, edinB, hla, hld, hlg, hlg2*	3	ST4723
S218	Cat owner	12/02/2018	Nasal	Bejaia	PNG1	-	+	-	*clfB, fib, sea, sek, sei, seg, seq, sed, hla, hld, hlg, hlg2*	1	ST217
S220	Dog owner	21/04/2018	Nasal	Bejaia (El Kseur)	-	-	+	-	*fnbB, clfB, fib, sea, sei, seg, seh, hla, hld, hlg, hlg2*	1	ST6
S222	Dog owner	22/04/2018	Nasal	Bejaia	PNG1	-	+	-	*clfB, sei, seg, hld, hlg2*	1	ST30
S223	Dog owner	22/04/2018	Nasal	Bejaia (Amizour)	PNG1	-	+	-	*clfB, sei, seg, hla, hld, hlg2*	2	ST30
S228	Dog owner	17/04/2018	Nasal	Bejaia	-	-	+	-	*fnbA, clfB, fib, sea, sek, sei, seh, hla, hlb, hld, hlg, hlg2*	1	ST6
S232	Dog owner	22/04/2018	Nasal	Bejaia	PNG1	-	+	-	*clfB, fib, sei, seg, hla, hlb, hld, hlg2*	3	ST30
S233	Farmer	17/02/2018	Nasal	Bousaada	PNG1	-	+	-	*fnbB, clfB, fib, seb, sei, edinB, hla, hld, hlg, hlg2*	1	ST97
S238	Farmer	28/04/2018	Nasal	Bejaia (Beni Maouche)	PNG1	-	+	+	*fnbA, clfB, fib, etb, sea, seb, sei, seg, hla, hld, hlg, hlg2*	1	ST97
S247	Human (Community)	10/05/2018	Nasal	Boumerdes	PNG1	-	+	-	*fnbB, clfB, fib, sea, sek, sei, seg, seq, seh, hla, hld, hlg, hlg2*	1	ST199
S248	Human (Community)	24/02/2018	Nasal	Bejaia (El Kseur)	PNG1	-	+	-	*fnbB, clfB, fib, sea, sek, sei, seg, seq, seh, hla, hld, hlg, hlg2*	1	ST573
S249	Human (Community)	29/03/2018	Nasal	Bejaia (Oued Ghir)	PNG1	-	+	-	*fnbB, clfB, fib, sea, sek, sei, seg, seq, seh, hla, hld, hlg, hlg2*	1	ST1
S256	Human (Community)	01/02/2018	Nasal	Setif	PNG1	-	+	-	*clfB, fib, sei, seg, seh, hla, hld, hlg, hlg2*	3	ST34
S258	Human (Community)	13/01/2018	Nasal	Setif	PNG1	-	+	-	*fnbB, clfB, sea, sei, seg, edinB, hld, hlg2*	1	ST291

S259	Human (Community)	16/01/2018	Nasal	Setif	PNG1	-	+	-	*clfB, sea, sei, seg, hld, hlg2*	3	ST30
S260	Human (Community)	02/05/2018	Nasal	Tizi Ouzou	PNG1	-	+	-	*clfB, fib, sei, seg, seh, hla, hld, hlg2*	1	ST22
S262	Human (Community)	17/02/2018	Nasal	Bousaada	PNG1	-	+	-	*clfB, sec, sei, seg, hla, hld, hlg2*	1	ST22
S263	Human (Community)	10/05/2018	Nasal	Boumerdes	PNG1	-	+	-	*fnbB, clfB, fib, sei, seg, hla, hlb, hld, hlg, hlg2*	3	ST30
S264	Human (Community)	01/05/2018	Nasal	Tizi Ouzou	PNG1	-	+	+	*fnbB, clfB, fib, eta, sea, sei, seg, hla, hlb, hld, hlg, hlg2*	3	ST4685
S265	Human (Community)	10/05/2018	Nasal	Boumerdes	PNG1	-	+	-	*fnbA, clfB, fib, sea, sei, seg, hla, hld, hlg, hlg2*	3	ST30
S279	Cow milk	06/02/2018	Milk	Bejaia (Boukhelifa)	PNG1-KMN-TMN	-	+	-	*clfB, fib, seb, sec, sei, edinB, hla, hld, hlg, hlg2*	1	ST7
S281	Goat milk	11/02/2018	Milk	Bejaia (Sidi Aich)	-	-	+	-	*fnbB, clfB, fib, sea, sec, sei, edinB, hla, hlb, hld, hlg, hlg2*	1	ST6
S287	Table egg	02/03/2018	Surface swabbing	Algiers (Baraki)	-	-	+	-	*fnbB, clfB, fib, seb, sek, sei, seg, seq, hla, hld, hlg, hlg2*	1	ST97
S95	Rabbit	02/05/2018	Nasal	Tizi Ouzou	PNG1	-	-	+	*fnbA, clfB, fib, etb, sea, sei, hla, hld, hlg, hlg2*	1	ST6
S102	Laying hens	02/05/2018	Rectal	Tizi Ouzou	PNG1	-	-	+	*clfB, fib, etb, sea, sei, seg, hla, hld, hlg, hlg2*	1	ST97
S104	Laying hens	26/04/2018	Rectal	Bejaia (Akbou)	-	-	-	+	*clfB, fib, etb, sea, sei, seg, hla, hld, hlg, hlg2*	1	ST6
S107	Laying hens	26/04/2018	Rectal	Bejaia (Akbou)	PNG1	-	-	+	*clfB, fib, eta, sei, seg, hla, hld, hlg, hlg2*	4	ST121
S108	Laying hens	17/02/2018	Rectal	Bousaada	PNG1	-	-	+	*fnbB, clfB, fib, eta, sea, sei, seg, hla, hld, hlg, hlg2*	1	ST398
S109	Laying hens	17/02/2018	Rectal	Bousaada	PNG1	-	-	+	*clfB, fib, etb, sea, sei, seg, hla, hld, hlg, hlg2*	1	ST6
S111	Laying hens	28/04/2018	Rectal	Bejaia (Beni Maouche)	PNG1	-	-	+	*clfB, fib, etb, sei, seg, hla, hld, hlg, hlg2*	4	ST121
S115	Laying hens	28/04/2018	Rectal	Bejaia (Ighil Ali)	PNG1	-	-	+	*fnbB, clfB, fib, etb, sea, sei, seg, hla, hld, hlg, hlg2*	1	ST398
S122	Laying hens	26/04/2018	Oral	Bejaia (Akbou)	PNG1	-	-	+	*fnbB, clfB, fib, eta, sea, seb, sei, seg, hla, hld, hlg, hlg2*	1	ST398
S124	Laying hens	18/04/2018	Rectal	Bejaia (Amizour)	PNG1	-	-	+	*clfB, fib, etb, sea, sei, seg, hla, hld, hlg, hlg2*	1	ST6
S125	Laying hens	02/05/2018	Oral	Tizi Ouzou	PNG1	-	-	+	*clfB, fib, etb, sea, sei, seg, hla, hld, hlg, hlg2*	1	ST6

S126	Laying hens	19/02/2018	Rectal	Bejaia (Melbou)	PNG1	-	-	+	*clfB, fib, etb, sea, sei, seg, hla, hld, hlg, hlg2*	1	ST6
S127	Laying hens	28/04/2018	Oral	Bejaia (Ighil Ali)	PNG1	-	-	+	*clfB, fib, etb, sea, sei, seg, hla, hld, hlg, hlg2*	1	ST5
S136	Laying hens	18/04/2018	Oral	Bejaia (Amizour)	PNG1	-	-	+	*fnbA, clfB, fib, etb, sea, sei, seg, hla, hld, hlg, hlg2*	1	ST6
S139	Laying hens	28/04/2018	Oral	Bejaia (Ighil Ali)	PNG1	-	-	+	*clfB, fib, etb, sei, seg, hla, hld, hlg, hlg2*	2	ST15
S140	Laying hens	28/04/2018	Oral	Bejaia (Beni Maouche)	PNG1	-	-	+	*clfB, fib, etb, sea, sei, seg, hla, hld, hlg, hlg2*	1	ST5
S143	Laying hens	28/04/2018	Rectal	Bejaia (Beni Maouche)	-	-	-	+	*clfB, fib, etb, sea, seg, hla, hld, hlg*	1	ST194
S144	Laying hens	28/04/2018	Oral	Bejaia (Ighil Ali)	-	-	-	+	*clfB, fib, etb, sea, sei, seg, hla, hld, hlg, hlg2*	1	ST5
S146	Laying hens	02/03/2018	Rectal	Algiers (Baraki)	-	-	-	+	*fnbA, clfB, fib, etb, sea, sek, sei, seg, seq, hla, hld, hlg, hlg2*	1	ST97
S147	Laying hens	18/04/2018	Oral	Bejaia (Amizour)	-	-	-	+	*clfB, fib, etb, sea, sei, seg, hla, hld, hlg, hlg2*	1	ST291
S149	Laying hens	18/04/2018	Oral	Bejaia (Amizour)	-	-	-	+	*clfB, fib, etb, sea, sei, seg, hla, hld, hlg, hlg2*	1	ST15
S150	Laying hens	26/04/2018	Oral	Bejaia (Akbou)	PNG1	-	-	+	*clfB, fib, eta, sei, seg, hla, hld, hlg, hlg2*	2	ST15
S161	Broilers	18/02/2018	Rectal	Bejaia (El Kseur)	ERY, iMLSb	-	-	+	*clfB, fib, etb, sei, seg, hla, hld, hlg, hlg2*	4	ST121
S226	Dog owner	25/04/2018	Nasal	Bejaia	PNG1	-	-	+	*fnbA, clfB, fib, etb, sea, sei, seg, hla, hld, hlg, hlg2*	1	ST199
S245	Human (Community)	11/07/2018	Nasal	Bouira	PNG1-KMN	-	-	+	*fnbB, clfB, fib, eta, sei, hla, hld, hlg, hlg2*	2	ST151
S268	Human (Community)	17/02/2018	Nasal	Bousaada	PNG1	-	-	+	*fnbA, clfB, fib, eta, sec, sei, seg, hla, hld, hlg, hlg2*	3	ST4685
S289	Table egg	02/03/2018	Surface swabbing	Algiers (Baraki)	-	-	-	+	*clfB, fib, etb, sea, sei, seg, hla, hld, hlg, hlg2*	1	ST7
S1	Wild bird (*Columba livia*)	04/03/2018	Feces	Bejaia	PNG1	-	-	-	*clfB, fib, sec, sei, seg, hld, hlg2*	1	NT
S2	Wild bird (*Columba livia*)	29/05/2018	Feces	Setif	RIF	-	-	-	*clfB, fib, sea, sei, hla, hld, hlg, hlg2*	1	NT
S3	Wild bird (*Columba livia*)	29/05/2018	Feces	Setif	PNG1-ERY, iMLSb	-	-	-	*fnbB, clfB, fib, sea, sei, hla, hld, hlg, hlg2*	1	NT
S4	Wild bird (*Columba livia*)	18/04/2018	Feces	Bejaia	PNG1	-	-	-	*clfB, fib, sec, sei, seg, hld, hlg2*	1	NT

S5	Wild bird (*Columba livia*)	04/03/2018	Feces	Bejaia	PNG1	-	-	-	*fnbB, clfB, fib, sea, sec, sei, seg, hla, hld, hlg, hlg2*	1	NT
S6	Wild bird (*Columba livia*)	04/03/2018	Feces	Bejaia	PNG1	-	-	-	*fnbB, clfB, fib, sei, hla, hld, hlg, hlg2*	2	NT
S7	Wild bird (*Columba livia*)	22/04/2018	Feces	Bejaia	PNG1-RIF	-	-	-	*clfB, cna, sea, sei, hla, hld, hlg, hlg2*	2	NT
S8	Wild bird (*Columba livia*)	18/04/2018	Feces	Bejaia	PNG1	-	-	-	*clfB, cna, sea, sei, hla, hld, hlg, hlg2*	2	NT
S9	Wild bird (*Columba livia*)	24/04/2018	Feces	Bejaia	-	-	-	-	*clfB, cna, sea, sei, hla, hld, hlg, hlg2*	2	NT
S10	Wild bird (*Columba livia*)	18/04/2018	Feces	Bejaia	PNG1	-	-	-	*clfB, fib, sea, sei, hla, hld, hlg, hlg2*	1	NT
S11	Wild bird (*Columba livia*)	30/06/2018	Feces	Mila	-	-	-	-	*clfB, fib, sea, sei, hla, hld, hlg, hlg2*	1	NT
S12	Wild bird (*Columba livia*)	24/05/2018	Feces	Jijel	PNG1	-	-	-	*clfB, fib, sea, sei, hla, hld, hlg, hlg2*	1	NT
S13	Wild bird (*Columba livia*)	24/05/2018	Feces	Jijel	PNG1	-	-	-	*clfB, fib, sea, sei, hla, hld, hlg, hlg2*	1	NT
S14	Wild bird (*Columba livia*)	30/06/2018	Feces	Mila	-	-	-	-	*clfB, fib, sea, sei, hla, hld, hlg, hlg2*	1	NT
S15	Wild bird (*Anas platyrhynchos*)	14/01/2018	Feces	Jijel	-	-	-	-	*clfB, fib, hla, hlb, hld, hlg*	1	NT
S16	Boar	07/05/2018	Nasal	Bejaia (Toudja)	-	-	-	-	*clfB, fib, sea, sei, hla, hlb, hld, hlg, hlg2*	1	NT
S17	Boar	03/03/2018	Nasal	Bejaia (Barbacha)	PNG1	-	-	-	*clfB, fib, sea, sei, hla, hld, hlg, hlg2*	1	NT
S18	Boar	03/03/2018	Nasal	Bejaia (Barbacha)	-	-	-	-	*clfB, cna, sea, sei, hla, hld, hlg, hlg2*	1	NT
S19	Boar	07/05/2018	Nasal	Bejaia (Toudja)	PNG1	-	-	-	*clfB, cna, sea, sei, hla, hld, hlg, hlg2*	1	NT
S20	Boar	10/02/2018	Nasal	Bejaia (Ikoubab)	-	-	-	-	*clfB, fib, sei, hla, hlb, hld, hlg, hlg2*	2	NT
S21	Wild fish (*Sardina pilchardus*)	30/04/2018	Intestine	Bejaia	PNG1	-	-	-	*clfB, fib, sei, hla, hld, hlg, hlg2*	1	NT
S22	Wild fish (*Sardina pilchardus*)	14/03/2018	Intestine	Jijel	PNG1	-	-	-	*clfB, fib, sea, sec, seq, seh, hla, hld, hlg, hlg2*	3	NT
S23	Wild fish (*Trachurus trachurus*)	14/03/2018	Intestine	Jijel	PNG1	-	-	-	*fnbB, clfB, fib, sei, hla, hld, hlg, hlg2*	2	NT
S24	Wild fish (*Mullus surmuletus*)	03/01/2018	Intestine	Bejaia	PNG1	-	-	-	*clfB, fib, sea, sek, seq, seh, hla, hld, hlg, hlg2*	3	NT

S25	Wild fish (*Sarpa sarpa*)	25/04/2018	Intestine	Jijel	-	-	-	-	*clfB, fib, sei, hla, hlb, hld, hlg, hlg2*	1	NT
S26	Wild fish (*Merlangius merlangus*)	03/01/2018	Intestine	Bejaia	PNG1-FAD	-	-	-	*clfB, fib, sea, sek, seq, seh, hla, hld, hlg, hlg2*	3	NT
S27	Wild fish (*Pagellus bogaraveo*)	11/07/2018	Intestine	Tizi Ouzou	PNG1-FAD	-	-	-	*clfB, fib, sea, sec, seq, seh, hla, hld, hlg, hlg2*	3	NT
S28	Monkey (*Macaca sylvanus*)	25/04/2018	Feces	Bejaia (Gouraya)	PNG1	-	-	-	*clfB, fib, sea, sei, hla, hld, hlg, hlg2*	1	NT
S29	Porcupine (*Hystrix cristata*)	20/04/2018	Nasal	Bejaia (Barbacha)	-	-	-	-	*clfB, fib, sei, hla, hlb, hld, hlg, hlg2*	2	NT
S32	Bovine (*Bos taurus*)	17/02/2018	Nasal	Bousaada	-	-	-	-	*fnbB, clfB, fib, hla, hlb, hld, hlg*	1	NT
S33	Bovine (*Bos taurus*)	30/06/2018	Nasal	Mila	PNG1-KMN	-	-	-	*fnbB, clfB, fib, seb, sei, hla, hld, hlg, hlg2*	1	NT
S34	Bovine (*Bos taurus*)	06/02/2018	Nasal	Constantine	PNG1	-	-	-	*fnbB, clfB, fib, seb, sei, seg, edinB, hla, hlb, hld, hlg, hlg2*	1	NT
S35	Bovine (*Bos taurus*)	22/04/2018	Nasal	Bourdj Bou Arreridj (Zemoura)	PNG1	-	-	-	*clfB, fib, sea, sei, hla, hld, hlg, hlg2*	1	NT
S36	Bovine (*Bos taurus*)	22/04/2018	Nasal	Bourdj Bou Arreridj (Ouled Bounem)	PNG1	-	-	-	*fnbB, clfB, fib, sea, sei, hla, hld, hlg, hlg2*	1	NT
S37	Bovine (*Bos taurus*)	22/04/2018	Nasal	Bourdj Bou Arreridj (Zemoura)	-	-	-	-	*clfB, fib, hla, hlb, hld, hlg2*	1	NT
S39	Bovine (*Bos taurus*)	13/02/2018	Nasal	Bejaia (Oued Ghir)	PNG1	-	-	-	*clfB, fib, sei, seg, hla, hld, hlg, hlg2*	2	NT
S40	Bovine (*Bos taurus*)	06/02/2018	Nasal	Bejaia (Boukhelifa)	-	-	-	-	*fnbB, clfB, fib, seb, sei, hla, hld, hlg, hlg2*	1	NT
S41	Bovine (*Bos taurus*)	13/02/2018	Nasal	Bejaia (Beni Maouche)	-	-	-	-	*fnbB, clfB, fib, sei, hla, hlb, hld, hlg, hlg2*	1	NT
S42	Bovine (*Bos taurus*)	22/04/2018	Nasal	Bourdj Bou Arreridj (Khelil)	PNG1	-	-	-	*clfB, fib, sea, sei, hla, hld, hlg, hlg2*	1	NT
S43	Bovine (*Bos taurus*)	22/04/2018	Nasal	Bourdj Bou Arreridj (Khelil)	PNG1	-	-	-	*clfB, cna, sea, sei, hla, hld, hlg, hlg2*	1	NT
S44	Bovine (*Bos taurus*)	22/04/2018	Nasal	Bourdj Bou Arreridj (Khelil)	PNG1	-	-	-	*fnbB, clfB, fib, sea, sei, hla, hld, hlg, hlg2*	1	NT
S45	Bovine (*Bos taurus*)	22/04/2018	Nasal	Bourdj Bou Arreridj (Zemoura)	PNG1	-	-	-	*fnbB, clfB, fib, sea, sei, hla, hld, hlg, hlg2*	1	NT
S48	Ovine (*Ovis aries*)	06/02/2018	Nasal	Bejaia (Boukhelifa)	-	-	-	-	*fnbB, clfB, fib, sei, seg, hla, hld, hlg, hlg2*	1	NT
S49	Ovine (*Ovis aries*)	12/02/2018	Nasal	Bejaia (Ighezar Amokrane)	-	-	-	-	*fnbB, clfB, fib, seb, sei, hla, hld, hlg, hlg2*	1	NT

S50	Ovine (*Ovis aries*)	30/06/2018	Nasal	Mila	-	-	-	-	*fnbB, clfB, cna, sei, seg, hla, hlb, hld, hlg, hlg2*	1	NT
S52	Ovine (*Ovis aries*)	06/02/2018	Nasal	Bejaia (Boukhelifa)	PNG1-KMN	-	-	-	*fnbB, clfB, fib, sei, hla, hld, hlg, hlg2*	1	NT
S53	Ovine (*Ovis aries*)	11/07/2018	Nasal	Bouira	PNG1	-	-	-	*fnbB, clfB, fib, sea, sei, edinB, hla, hld, hlg, hlg2*	1	NT
S55	Ovine (*Ovis aries*)	01/02/2018	Nasal	Setif	PNG1	-	-	-	*fnbB, clfB, fib, sei, seg, hla, hld, hlg, hlg2*	2	NT
S58	Ovine (*Ovis aries*)	22/04/2018	Nasal	Bourdj Bou Arreridj (Zemoura)	PNG1	-	-	-	*clfB, fib, sea, sei, hla, hld, hlg, hld2*	1	NT
S59	Ovine (*Ovis aries*)	22/04/2018	Nasal	Bourdj Bou Arreridj (Zemoura)	PNG1	-	-	-	*clfB, fib, sea, sei, hla, hld, hlg*	1	NT
S61	Ovine (*Ovis aries*)	17/04/2018	Nasal	Bejaia (Amizour)	PNG1	-	-	-	*fnbB, clfB, fib, sea, sei, hla, hld, hlg, hlg2*	1	NT
S64	Ovine (*Ovis aries*)	12/02/2018	Nasal	Bejaia (Akbou)	-	-	-	-	*fnbB, clfB, fib, sec, sei, seg, seh, edinB, hla, hlb, hld, hlg, hlg2*	1	NT
S66	Ovine (*Ovis aries*)	01/02/2018	Nasal	Setif	PNG1	-	-	-	*clfB, fib, seb, sei, hla, hld, hlg, hlg2*	1	NT
S67	Ovine (*Ovis aries*)	17/02/2018	Nasal	Bousaada	-	-	-	-	*fnbB, clfB, cna, sea, sei, hla, hld, hlg, hlg2*	1	NT
S68	Ovine (*Ovis aries*)	17/02/2018	Nasal	Bousaada	-	-	-	-	*clfB, fib, sei, hla, hlb, hld, hlg, hlg2*	1	NT
S69	Ovine (*Ovis aries*)	24/05/2018	Nasal	Jijel	PNG1-KMN	-	-	-	*fnbB, clfB, fib, seb, sei, hla, hld, hlg, hlg2*	1	NT
S70	Ovine (*Ovis aries*)	24/05/2018	Nasal	Jijel	PNG1-KMN	-	-	-	*fnbB, clfB, fib, seb, sei, hla, hld, hlg, hlg2*	1	NT
S71	Ovine (*Ovis aries*)	01/02/2018	Nasal	Setif	PNG1-ERY, iMLSb	-	-	-	*fnbB, clfB, fib, sei, hld, hlg, hlg2*	1	NT
S72	Ovine (*Ovis aries*)	02/05/2018	Nasal	Tizi Ouzou	PNG1	-	-	-	*fnbB, clfB, fib, sei, hla, hlb, hld, hlg, hlg2*	1	NT
S81	Goat (*Capra hircus*)	22/05/2018	Nasal	Bourdj Bou Arreridj (Khelil)	PNG1	-	-	-	*clfB, cna, sea, sei, seh, hla, hld, hlg, hlg2*	1	NT
S82	Goat (*Capra hircus*)	22/05/2018	Nasal	Bourdj Bou Arreridj (Khelil)	-	-	-	-	*clfB, fib, sei, seh, hla, hlb, hld, hlg, hlg2*	3	NT
S83	Goat (*Capra hircus*)	06/02/2018	Nasal	Bejaia (Boukhelifa)	-	-	-	-	*fnbA, clfB, fib, sea, sec, sei, seg, edinB, hla, hlb, hld, hlg, hlg2*	1	NT
S84	Goat (*Capra hircus*)	02/05/2018	Nasal	Tizi Ouzou	-	-	-	-	*fnbB, clfB, fib, sec, seh, edinB, hla, hlb, hld, hlg, hlg2*	1	NT
S85	Goat (*Capra aegagrus hircus*)	22/04/2018	Nasal	Bourdj Bou Arreridj (Zemoura)	PNG1	-	-	-	*fnbB, clfB, fib, sei, hla, hld, hlg, hlg2*	2	NT
S86	Goat (*Capra aegagrus hircus*)	22/04/2018	Nasal	Bourdj Bou Arreridj (Zemoura)	PNG1	-	-	-	*fnbB, clfB, fib, sei, hla, hld, hlg, hlg2*	2	NT

S87	Goat (*Capra aegagrus hircus*)	02/05/2018	Nasal	Tizi Ouzou	PNG1	-	-	-	*clfB, fib, sea, sei, hla, hld, hlg, hlg2*	1	NT
S89	Rabbit	18/04/2018	Nasal	Bejaia (Amizour)	-	-	-	-	*fnbB, clfB, fib, sei, hla, hld, hlg, hlg2*	2	NT
S90	Rabbit	18/04/2018	Nasal	Bejaia (Amizour)	-	-	-	-	*clfB, fib, sea, sei, hla, hld, hlg, hlg2*	1	NT
S91	Rabbit	18/04/2018	Nasal	Bejaia (Amizour)	-	-	-	-	*clfB, cna, sei, hla, hlb, hld, hlg, hlg2*	1	NT
S93	Rabbit	09/02/2018	Nasal	Bejaia (Ouzellaguen)	PNG1	-	-	-	*fnbB, clfB, fib, sei, hla, hld, hlg, hlg2*	2	NT
S96	Rabbit	25/04/2018	Nasal	Bejaia (Barbacha)	-	-	-	-	*fnbB, clfB, fib, sec, sei, seg, seh, hla, hlb, hld, hlg, hlg2*	3	NT
S97	Rabbit	18/04/2018	Nasal	Bejaia (Amizour)	-	-	-	-	*fnbB, clfB, fib, sei, seh, hla, hlb, hld, hlg, hlg2*	1	NT
S98	Rabbit	18/04/2018	Nasal	Bejaia (Amizour)	-	-	-	-	*fnbB, clfB, fib, sei, seh, edinB, hla, hlb, hld, hlg, hlg2*	3	NT
S99	Rabbit	18/04/2018	Nasal	Bejaia (Amizour)	-	-	-	-	*fnbB, clfB, fib, hla, hlb, hld, hlg2*	1	NT
S100	Rabbit	04/05/2018	Nasal	Bejaia (Kherrata)	PNG1	-	-	-	*clfB, fib, sec, sei, seg, hld, hlg2*	1	NT
S101	Laying hens	02/03/2018	Oral	Algiers (Baraki)	PNG1	-	-	-	*clfB, fib, sea, sei, hla, hld, hlg, hlg2*	1	NT
S103	Laying hens	26/04/2018	Oral	Bejaia (Akbou)	-	-	-	-	*fnbB, clfB, fib, sea, sec, sek, sei, seg, seq, seh, hla, hld, hlg, hlg2*	1	NT
S105	Laying hens	28/04/2018	Oral	Bejaia (Beni Maouche)	PNG1	-	-	-	*clfB, fib, sea, sei, hla, hld, hlg, hlg2*	1	NT
S106	Laying hens	04/05/2018	Rectal	Bejaia (Kherrata)	PNG1	-	-	-	*clfB, fib, sec, sei, seg, hld, hlg2*	1	NT
S110	Laying hens	16/04/2018	Rectal	Bejaia (Timezrit)	PNG1	-	-	-	*clfB, fib, sea, sei, hla, hld, hlg, hlg2*	1	NT
S112	Laying hens	17/02/2018	Oral	Bousaada	PNG1	-	-	-	*clfB, fib, sea, sei, hla, hld, hlg, hlg2*	1	NT
S113	Laying hens	02/03/2018	Rectal	Algiers (Baraki)	PNG1	-	-	-	*fnbA, clfB, fib, hla, hld, hlg, hlg2*	3	NT
S114	Laying hens	28/04/2018	Oral	Bejaia (Beni Maouche)	PNG1	-	-	-	*clfB, fib, sea, sei, hla, hld, hlg, hlg2*	1	NT
S116	Laying hens	22/04/2018	Oral	Bourdj Bou Arreridj (Khelil)	-	-	-	-	*fnbA, clfB, fib, sea, sei, edinB, hla, hld, hlg, hlg2*	1	NT
S118	Laying hens	21/04/2018	Oral	Bejaia (Beni Ksila)	PNG1-KMN	-	-	-	*fnbB, clfB, fib, seb, sei, hla, hld, hlg, hlg2*	1	NT
S119	Laying hens	18/04/2018	Oral	Bejaia (Amizour)	PNG1	-	-	-	*clfB, fib, sea, sei, hla, hld, hlg, hlg2*	1	NT
S120	Laying hens	16/04/2018	Rectal	Bejaia (Timezrit)	PNG1	-	-	-	*clfB, fib, sea, sei, seg, hla, hld, hlg, hlg2*	1	NT

S121	Laying hens	02/03/2018	Rectal	Algiers (Baraki)	PNG1	-	-	-	*clfB, fib, sea, sei, seg, hla, hld, hlg, hlg2*	1	NT
S123	Laying hens	28/04/2018	Oral	Bejaia (Ighil Ali)	PNG1	-	-	-	*clfB, fib, sea, sei, seg, hla, hld, hlg, hlg2*	1	NT
S128	Laying hens	16/04/2018	Oral	Bejaia (Timezrit)	PNG1	-	-	-	*clfB, fib, sea, sei, hla, hld, hlg, hlg2*	1	NT
S129	Laying hens	26/04/2018	Oral	Bejaia (Akbou)	PNG1	-	-	-	*clfB, fib, sea, sei, hla, hld, hlg, hlg2*	1	NT
S130	Laying hens	26/04/2018	Rectal	Bejaia (Akbou)	PNG1	-	-	-	*fnbA, clfB, fib, sea, sei, edinB, hla, hld, hlg, hlg2*	1	NT
S131	Laying hens	18/04/2018	Rectal	Bejaia (Amizour)	PNG1	-	-	-	*clfB, fib, sea, sek, seq, hla, hld, hlg, hlg2*	1	NT
S132	Laying hens	19/02/2018	Oral	Bejaia (Melbou)	PNG1	-	-	-	*clfB, fib, sea, sei, hla, hld, hlg, hlg2*	1	NT
S133	Laying hens	18/04/2018	Rectal	Bejaia (Amizour)	PNG1	-	-	-	*fnbA, clfB, fib, sea, sek, seq, hla, hld, hlg, hlg2*	1	NT
S134	Laying hens	16/04/2018	Rectal	Bejaia (Timezrit)	PNG1	-	-	-	*clfB, fib, sea, sek, sek, hla, hld, hlg, hlg2*	1	NT
S137	Laying hens	26/04/2018	Oral	Bejaia (Akbou)	PNG1	-	-	-	*clfB, fib, sea, sek, seq, hla, hld, hlg, hlg2*	1	NT
S141	Laying hens	02/03/2018	Rectal	Algiers (Baraki)	PNG1	-	-	-	*fnbA, clfB, fib, sea, sek, seq, hla, hld, hlg, hlg2*	1	NT
S142	Laying hens	19/02/2018	Oral	Bejaia (Melbou)	-	-	-	-	*clfB, fib, sea, sei, hla, hld, hlg, hlg2*	1	NT
S148	Laying hens	19/02/2018	Rectal	Bejaia (Melbou)	-	-	-	-	*fnbB, clfB, fib, sea, sei, hla, hld, hlg, hlg2*	1	NT
S151	Laying hens	16/04/2018	Rectal	Bejaia (Timezrit)	PNG1	-	-	-	*clfB, fib, sea, sei, hla, hld, hlg, hlg2*	1	NT
S153	Laying hens	19/05/2018	Oral	Batna	-	-	-	-	*clfB, fib, sea, sei, hla, hld, hlg, hlg2*	1	NT
S154	Laying hens	26/04/2018	Oral	Bejaia (Akbou)	PNG1	-	-	-	*clfB, fib, sea, sei, hla, hld, hlg, hlg2*	1	NT
S159	Broilers	18/02/2018	Oral	Bejaia (El Kseur)	ERY-MNO, iMLSb	-	-	-	*clfB, fib, sei, seg, hla, hlb, hld, hlg, hlg2*	2	NT
S160	Broilers	19/05/2018	Rectal	Batna	PNG1-ERY, iMLSb	-	-	-	*clfB, fib, sea, sei, seg, hla, hlb, hld, hlg, hlg2*	2	NT
S162	Turkey	25/04/2018	Oral	Bejaia (Timezrit)	PNG1	-	-	-	*clfB, fib, sea, sei, hla, hld, hlg, hlg2*	1	NT
S163	Turkey	19/02/2018	Rectal	Algiers (Salembier)	PNG1-ERY, iMLSb	-	-	-	*clfB, fib, sei, hla, hlb, hld, hlg, hlg2*	1	NT

S164	Turkey	19/02/2018	Oral	Algiers (Salembier)	PNG1	-	-	-	*clfB, fib, seb, sei, hla, hld, hlg, hlg2*	1	NT
S165	Turkey	19/02/2018	Rectal	Algiers (Salembier)	-	-	-	-	*fnbB, clfB, fib, sea, seb, sek, seq, hla, hlb, hld, hlg, hlg2*	1	NT
S166	Turkey	19/02/2018	Oral	Algiers (Salembier)	-	-	-	-	*fnbB, clfB, fib, sea, sei, hla, hlb, hld, hlg, hlg2*	1	NT
S167	Cat	06/02/2018	Nasal	Bejaia	PNG1-ERY, iMLSb	-	-	-	*clfB, fib, sei, hla, hlb, hld, hlg, hlg2*	1	NT
S168	Cat	21/04/2018	Nasal	Bejaia (Amizour)	PNG1	-	-	-	*clfB, fib, sea, sei, hla, hlb, hld, hlg, hlg2*	1	NT
S169	Cat	20/04/2018	Nasal	Bejaia (El Kseur)	PNG1	-	-	-	*clfB, fib, sea, sei, hla, hld, hlg, hlg2*	1	NT
S170	Cat	19/05/2018	Nasal	Batna	-	-	-	-	*fnbB, clfB, fib, sea, sei, hla, hld, hlg, hlg2*	1	NT
S171	Cat	04/03/2018	Nasal	Bejaia (Sidi Aich)	-	-	-	-	*fnbB, clfB, fib, sea, sei, hla, hlb, hld, hlg, hlg2*	3	NT
S172	Cat	04/03/2018	Nasal	Bejaia (Sidi Aich)	-	-	-	-	*fnbA, clfB, fib, sea, sei, seg, seh, hla, hlb, hld, hlg, hlg2*	1	NT
S174	Cat	10/05/2018	Nasal	Boumerdes	PNG1	-	-	-	*clfB, fib, sea, sei, hla, hld, hlg, hlg2*	1	NT
S175	Cat	21/04/2018	Nasal	Bejaia (El Kseur)	PNG1	-	-	-	*clfB, fib, sea, sek, sei, seg, seq, sed, hla, hld, hlg, hlg2*	1	NT
S176	Cat	04/03/2018	Nasal	Bejaia (Sidi Aich)	PNG1	-	-	-	*fnbA, clfB, fib, hla, hld, hlg, hlg2*	3	NT
S177	Cat	19/05/2018	Nasal	Batna	-	-	-	-	*clfB, fib, sea, sei, hla, hld, hlg, hlg2*	3	NT
S178	Cat	19/05/2018	Nasal	Batna	-	-	-	-	*clfB, fib, sei, hla, hlb, hld, hlg, hlg2*	1	NT
S179	Cat	20/04/2018	Nasal	Bejaia (El Kseur)	-	-	-	-	*clfB, fib, sea, sei, hla, hld, hlg, hlg2*	1	NT
S180	Cat	21/02/2018	Nasal	Bejaia	PNG1	-	-	-	*clfB, fib, seb, sei, hla, hld, hlg, hlg2*	1	NT
S182	Dog	24/04/2018	Nasal	Bejaia (El Kseur)	PNG1	-	-	-	*clfB, fib, sea, sei, hla, hld, hlg, hlg2*	1	NT
S183	Dog	19/05/2018	Nasal	Batna	PNG1	-	-	-	*clfB, fib, sei, hla, hlb, hld, hlg, hlg2*	1	NT
S184	Dog	19/05/2018	Nasal	Batna	PNG1	-	-	-	*fnbB, clfB, fib, sei, hla, hld, hlg, hlg2*	2	NT
S185	Dog	25/04/2018	Nasal	Bejaia	PNG1	-	-	-	*fnbB, clfB, fib, sea, sei, hla, hlb, hld, hlg, hlg2*	1	NT
S186	Dog	16/02/2018	Nasal	Algiers (Rouiba)	PNG1	-	-	-	*fnbB, clfB, fib, sei, hla, hld, hlg, hlg2*	2	NT

S188	Dog	17/04/2018	Nasal	Bejaia	PNG1	-	-	-	*fnbB, clfB, fib, sea, sei, hla, hld, hlg, hlg2*	1	NT
S189	Dog	21/04/2018	Nasal	Bejaia (Amizour)	PNG1	-	-	-	*fnbA, clfB, fib, sea, sek, hla, hld, hlg, hlg2*	1	NT
S190	Dog	10/05/2018	Nasal	Boumerdes	PNG1	-	-	-	*clfB, fib, sea, sei, hla, hld, hlg, hlg2*	3	NT
S191	Dog	10/05/2018	Nasal	Boumerdes	PNG1	-	-	-	*clfB, fib, sec, sei, seg, hld, hlg2*	1	NT
S192	Dog	05/03/2018	Nasal	Bejaia	PNG1	-	-	-	*fnbA, clfB, fib, sei, hla, hlb, hld, hlg, hlg2*	2	NT
S193	Dog	21/04/2018	Nasal	Bejaia (Amizour)	-	-	-	-	*clfB, fib, sea, sei, hla, hld, hlg, hlg2*	1	NT
S195	Dog	05/05/2018	Nasal	Mila	-	-	-	-	*fnbA, clfB, fib, sea, sek, sei, sed, hla, hld, hlg, hlg2*	1	NT
S196	Dog	17/04/2018	Nasal	Bejaia	-	-	-	-	*clfB, fib, sea, sei, hla, hld, hlg, hlg2*	1	NT
S197	Dog	26/04/2018	Nasal	Bejaia (Akbou)	-	-	-	-	*clfB, fib, sea, sei, hla, hld, hlg, hlg2*	3	NT
S198	Dog	18/02/2018	Nasal	Constantine	-	-	-	-	*clfB, fib, sea, sei, hla, hld, hlg, hlg2*	1	NT
S199	Dog	16/02/2018	Nasal	Algiers (Rouiba)	PNG1	-	-	-	*fnbB, clfB, fib, sei, hla, hld, hlg, hlg2*	1	NT
S201	Dog	16/02/2018	Nasal	Algiers (Rouiba)	PNG1	-	-	-	*fnbB, clfB, fib, sei, hla, hld, hlg, hlg2*	2	NT
S202	Dog	06/05/2018	Nasal	Bejaia (Barbacha)	-	-	-	-	*clfB, fib, sei, seg, hld, hlg2*	1	NT
S203	Dog	01/05/2018	Nasal	Tizi Ouzou	-	-	-	-	*clfB, fib, sei, hla, hld, hlg, hlg2*	1	NT
S204	Dog	05/05/2018	Nasal	Mila	-	-	-	-	*clfB, fib, sei, seg, hld, hlg2*	1	NT
S205	Horse	18/02/2018	Nasal	Constantine	PNG1	-	-	-	*clfB, fib, sea, sek, seq, sed, hla, hld, hlg, hlg2*	3	NT
S207	Horse	05/05/2018	Nasal	Bejaia (Adekar)	-	-	-	-	*fnbA, clfB, fib, sea, sec, sei, seh, hla, hlb, hld, hlg*	1	NT
S208	Horse	21/04/2018	Nasal	Bourdj Bou Arreridj (Chiffa)	PNG1	-	-	-	*clfB, fib, sea, sek, sed, hla, hlb, hld, hlg*	1	NT
S210	Horse	19/05/2018	Nasal	Batna	PNG1	-	-	-	*clfB, sei, seg, hla, hld, hlg2*	1	NT
S211	Horse owner	07/03/2018	Nasal	Algiers (Kharouba)	PNG1-ERY, iMLSb	-	-	-	*fnbB, clfB, fib, sei, hld, hlg2*	1	NT
S212	Horse owner	18/02/2018	Nasal	Constantine	PNG1	-	-	-	*clfB, fib, sei, hla, hlb, hld, hlg, hlg2*	2	NT
S219	Cat owner	13/02/2018	Nasal	Bejaia (Samaoune)	-	-	-	-	*clfB, fib, sea, sek, sei, seh, hla, hld, hlg, hlg2*	1	NT
S221	Dog owner	24/05/2018	Nasal	Jijel	-	-	-	-	*fnbB, clfB, fib, sea, sei, seg, seh, hla, hld, hlg, hlg2*	1	NT
S224	Dog owner	18/02/2018	Nasal	Constantine	PNG1	-	-	-	*clfB, fib, sec, sei, seg, hld, hlg2*	1	NT

S225	Dog owner	18/02/2018	Nasal	Constantine	PNG1	-	-	-	*fnbA, clfB, fib, sei, hla, hld, hlg, hlg2*	1	NT
S227	Dog owner	21/04/2018	Nasal	Bejaia (Amizour)	PNG1	-	-	-	*fnbA, clfB, fib, sea, sei, hla, hld, hlg, hlg2*	1	NT
S229	Dog owner	21/04/2018	Nasal	Bejaia (Amizour)	-	-	-	-	*fnbA, clfB, fib, sea, sei, hla, hld, hlg, hlg2*	3	NT
S230	Dog owner	15/04/2018	Nasal	Bejaia	-	-	-	-	*clfB, fib, sea, sei, hla, hld, hlg, hlg2*	2	NT
S231	Dog owner	11/07/2018	Nasal	Bouira	ERY, iMLSb	-	-	-	*fnbB, clfB, fib, sei, hla, hld, hlg, hlg2*	1	NT
S234	Farmer	24/05/2018	Nasal	Jijel	PNG1	-	-	-	*clfB, fib, sea, sei, hla, hld, hlg, hlg2*	1	NT
S235	Farmer	22/04/2018	Nasal	Bourdj Bou Arreridj (Khelil)	PNG1	-	-	-	*fnbB, clfB, fib, sea, sei, edinB, hld, hlg, hlg2*	1	NT
S236	Farmer	26/02/2018	Nasal	Bejaia (Akbou)	PNG1-ERY-CMN-OFX	-	-	-	*clfB, fib, sea, sek, seq, hla, hld, hlg, hlg2*	1	NT
S237	Farmer	19/02/2018	Nasal	Bejaia (Souk El Tenine)	PNG1	-	-	-	*fnbB, clfB, fib, sei, hla, hld, hlg, hlg2*	3	NT
S239	Farmer	26/02/2018	Nasal	Bejaia (Melbou)	PNG1-ERY-OFX	-	-	-	*clfB, fib, sea, sek, seq, hla, hld, hlg, hlg2*	1	NT
S240	Farmer	26/04/2018	Nasal	Bejaia (Akbou)	PNG1	-	-	-	*clfB, fib, sea, sei, hla, hld, hlg, hlg2*	1	NT
S241	Farmer	26/04/2018	Nasal	Bejaia (Akbou)	PNG1	-	-	-	*clfB, fib, sei, seg, sed, hla, hlb, hld, hlg2*	3	NT
S246	Human (Community)	02/05/2018	Nasal	Bejaia (Sidi Aich)	PNG1	-	-	-	*clfB, fib, sec, sei, seg, hld, hlg*	1	NT
S250	Human (Community)	19/05/2018	Nasal	Batna	PNG1-FAD	-	-	-	*clfB, fib, sea, sek, sei, seg, sek, seh, hla, hld, hlg, hlg2*	1	NT
S251	Human (Community)	19/05/2018	Nasal	Batna	PNG1	-	-	-	*clfB, fib, sec, sei, seg, hla, hld, hlg, hlg2*	1	NT
S252	Human (Community)	19/05/2018	Nasal	Batna	PNG1	-	-	-	*fnbA, clfB, fib, seb, sei, seg, hla, hlb, hld, hlg, hlg2*	2	NT
S253	Human (Community)	02/05/2018	Nasal	Tizi Ouzou	PNG1	-	-	-	*clfB, fib, sei, seg, hla, hld, hlg2*	1	NT
S254	Human (Community)	06/02/2018	Nasal	Constantine	-	-	-	-	*fnbB, clfB, fib, sei, hla, hld, hlg, hlg2*	1	NT
S255	Human (Community)	02/03/2018	Nasal	Algiers (Baraki)	PNG1	-	-	-	*fnbA, clfB, fib, sei, seg, edinB, hla, hld, hlg, hlg2*	2	NT
S257	Human (Community)	11/07/2018	Nasal	Bouira	PNG1	-	-	-	*fnbB, clfB, fib, sei, hla, hld, hlg, hlg2*	2	NT

S261	Human (Community)	11/07/2018	Nasal	Bouira	PNG1	-	-	-	*clfB, sei, seg, hla, hlb, hld, hlg2*	1	NT
S266	Human (Community)	06/02/2018	Nasal	Constantine	PNG1	-	-	-	*fnbA, clfB, fib, sei, seg, hla, hld, hlg, hlg2*	1	NT
S267	Human (Community)	17/02/2018	Nasal	Bousaada	PNG1	-	-	-	*fnbA, clfB, fib, sei, hla, hld, hlg, hlg2*	3	NT
S269	Meat	04/03/2018		Bejaia (Sidi Aich)	PNG1	-	-	-	*clfB, cna, sea, sei, hla, hld, hlg, hlg2*	1	NT
S270	Meat	26/02/2018		Bejaia	-	-	-	-	*clfB, cna, sei, hla, hld, hlg, hlg2*	1	NT
S271	Meat	11/07/2018		Bouira	PNG1-ERY, iMLSb	-	-	-	*fnbB, clfB, cna, sei, hla, hld, hlg, hlg2*	2	NT
S272	Meat	04/03/2018		Bejaia (Akbou)	-	-	-	-	*clfB, cna, sea, sei, hla, hlb, hld, hlg, hlg2*	1	NT
S273	Sausage	04/03/2018		Bejaia (Ighezar Amokrane)	-	-	-	-	*clfB, fib, sea, sei, hla, hlb, hld, hlg, hlg2*	1	NT
S274	Chicken offal's	16/06/2018		Bejaia (Sidi Aich)	PNG1-ERY, iMLSb	-	-	-	*fnbB, clfB, fib, hla, hld, hlg, hlg2*	2	NT
S275	Chicken offal's	10/05/2018		Boumerdes	PNG1-ERY, iMLSb	-	-	-	*fnbB, clfB, fib, sei, hla, hld, hlg, hlg2*	2	NT
S276	Cow milk	16/02/2018	Milk	Bejaia (Amizour)	PNG1-ERY, iMLSb	-	-	-	*fnbB, clfB, fib, sei, hla, hld, hlg, hlg2*	2	NT
S277	Cow milk	26/03/2018	Milk	Bouira	PNG1-ERY, iMLSb	-	-	-	*fnbB, clfB, fib, sei, hla, hld, hlg, hlg2*	2	NT
S278	Cow milk	01/04/2018	Milk	Bouira	PNG1-ERY-FAD, iMLSb	-	-	-	*fnbB, clfB, fib, hla, hld, hlg, hlg2*	2	NT
S280	Cow milk	02/05/2018	Milk	Tizi Ouzou	PNG1-KMN-TMN	-	-	-	*clfB, fib, sei, hla, hld, hlg, hlg2*	1	NT
S282	Goat milk	26/04/2018	Milk	Bejaia (Akbou)	-	-	-	-	*clfB, fib, sea, sei, hla, hld, hlg, hlg2*	1	NT
S283	Table egg	11/07/2018	Surface swabbing	Bouira	-	-	-	-	*fnbB, clfB, fib, sea, sei, seg, hla, hld, hlg, hlg2*	1	NT
S284	Table egg	11/07/2018	Surface swabbing	Bouira	-	-	-	-	*clfB, fib, sea, sei, hla, hld, hlg, hlg2*	1	NT
S285	Table egg	26/04/2018	Surface swabbing	Bejaia (Akbou)	-	-	-	-	*clfB, fib, sea, sei, hla, hld, hlg, hlg2*	1	NT
S286	Table egg	26/04/2018	Surface swabbing	Bejaia (Akbou)	-	-	-	-	*fnbB, clfB, fib, sea, sei, hla, hld, hlg, hlg2*	3	NT
S288	Table egg	02/05/2018	Surface swabbing	Tizi Ouzou	-	-	-	-	*fnbA, clfB, fib, sea, sei, hla, hld, hlg, hlg2*	1	NT
S290	Aquatic environment (River)	05/05/2018	Water	Jijel	-	-	-	-	*clfB, fib, sea, sei, seg, hla, hld, hlg, hlg2*	1	NT
S291	Aquatic environment (Sea)	10/05/2018	Water	Boumerdes	PNG1-KMN	-	-	-	*clfB, fib, sea, sei, hla, hld, hlg, hlg2*	1	NT

S292	Aquatic environment (River)	02/05/2018	Water	Tizi Ouzou	-	-	-	-	*clfB, fib, sea, sei, hla, hlb, hld, hlg, hlg2*	1	NT

Penicillin G1 (PNG1), clindamycin (CMN, 2 µg), erytromycin (ERY, 15 µg), fusidic acid (FAD, 10 µg), kanamycin (KMN, 30 µg), minocycline (MNO, 30 µg), ofloxacin (OFX, 5 µg), rifampicin (RIF, 5 µg), iMLSb (inducible macrolide-lincosamide-streptogramin B), TSST (Toxic Shock Syndrome Toxin), *agr* (accessory gene regulator). NT, not tested.
